# Raised Middle-Finger: Electrocortical Correlates of Social Conditioning with Nonverbal Affective Gestures

**DOI:** 10.1371/journal.pone.0102937

**Published:** 2014-07-23

**Authors:** Matthias J. Wieser, Tobias Flaisch, Paul Pauli

**Affiliations:** 1 Department of Psychology, University of Würzburg, Würzburg, Germany; 2 Department of Psychology, University of Konstanz, Konstanz, Germany; University of Tuebingen Medical School, Germany

## Abstract

Humans form impressions of others by associating persons (faces) with negative or positive social outcomes. This learning process has been referred to as social conditioning. In everyday life, affective nonverbal gestures may constitute important social signals cueing threat or safety, which therefore may support aforementioned learning processes. In conventional aversive conditioning, studies using electroencephalography to investigate visuocortical processing of visual stimuli paired with danger cues such as aversive noise have demonstrated facilitated processing and enhanced sensory gain in visual cortex. The present study aimed at extending this line of research to the field of social conditioning by pairing neutral face stimuli with affective nonverbal gestures. To this end, electro-cortical processing of faces serving as different conditioned stimuli was investigated in a differential social conditioning paradigm. Behavioral ratings and visually evoked steady-state potentials (ssVEP) were recorded in twenty healthy human participants, who underwent a differential conditioning procedure in which three neutral faces were paired with pictures of negative (raised middle finger), neutral (pointing), or positive (thumbs-up) gestures. As expected, faces associated with the aversive hand gesture (raised middle finger) elicited larger ssVEP amplitudes during conditioning. Moreover, theses faces were rated as to be more arousing and unpleasant. These results suggest that cortical engagement in response to faces aversively conditioned with nonverbal gestures is facilitated in order to establish persistent vigilance for social threat-related cues. This form of social conditioning allows to establish a predictive relationship between social stimuli and motivationally relevant outcomes.

## Introduction

Traditionally, in classical aversive conditioning either highly aversive electric stimuli [1], [2], [3], [4], [5] or loud aversive bursts of (white) noise [6], [7], [8], [9], [10] have been used as aversive unconditioned stimulus (US), which have been proven to elicit strong fear reactions and enhanced amygdala activity in response to the conditioned stimulus (CS). Comparable effects were found for other types of US, such as odor stimuli [11] and negative emotional pictures [12]. From a social neuroscience perspective however, one has to note that affective and social learning processes outside the laboratory are rarely happening with these types of US stimuli. In contrast, one may consider these types of stimuli as ecologically less valid because humans seldom encounter such stimuli in everyday life. Admittedly, social stimuli (verbal or non-verbal) are much more likely to function as US in everyday social learning situations, and thus contribute to impression formation and social and affective learning. Particularly, the ability to identify individual faces based on the social consequences they have predicted in the past constitutes an essential form of associative learning in humans. This learning mechanism has been coined social conditioning, defined as process whereby an individual learns to identify other individuals that have predicted threats or rewards in the past [13].

Only recently researchers have started using social and hence ecologically more valid US such as verbal descriptions (sentences), affective prosody, and facial expressions [13], [14], [15], [16] to investigate the effects and neural correlates of social conditioning. Using verbal feedback sentences as US (e.g., “He says you're stupid”), it was shown that faces associated with pleasant and unpleasant social outcomes elicited larger activations in the human amygdala compared to when subjects learned that a face predicted neutral social outcomes [13]. Consistent with these findings, pairing faces with aversive audiovisual US (negative faces combined with a male voice saying “Stupid”) also led to efficient social aversive learning and concurrent amygdala activation to the fear-associated CS face [14]. These studies suggests that social US, although less intense than conventional US, are sufficient to cause conditioning and modulate amygdala responses to previously neutral stimuli. In a further study in which social conditioning was investigated in social anxiety disorder, participants underwent differential social conditioning incorporating socially stressful US such as critical facial expressions combined with derogatory verbal feedback [15]. Interestingly, only socially anxious subjects demonstrated fear conditioning, as a potentiated startle blink reflex to the CS face predictive of a negative compared to both CS predictive of a neutral or a positive social outcome indicated. The latter study points at the notion that socially relevant US may especially disseminate their anxiogenic effect in individuals with social anxiety disorder. This notion was also recently supported by findings of enhanced amygdala activity in socially anxious individuals in response to neutral faces which have been previously associated with videos of negative feedback [16].

The aim of the current study was to examine the electrocortical correlates of social conditioning, i.e. how the visual brain responds to socially conditioned faces. The conditioned stimuli consisted of three neutral faces which were paired with unpleasant, neutral, or pleasant hand gestures during the acquisition phase. Symbolic hand gestures carrying affective meaning appear well-suited as social US, as they have been shown to be preferentially processed by the brain [17], [18], [19]. Steady-state visually evoked potentials (ssVEPs) in response to faces were used to quantify the degree of visuocortical engagement to the different CS cues. The ssVEP is an oscillatory response of the visual cortex elicited by luminance- or contrast-modulated stimuli in which the frequency of the electrocortical response recorded from the scalp equals that of the driving [20], [21]. Here, the frequency of the cortical response is precisely known and can therefore be reliably separated from noise and quantified in the frequency domain [22]. Moreover and of significant advantage in conditioning paradigms where the trial number is usually limited, ssVEPs possess excellent signal-to-noise ratios compared to traditional ERP components [23]. An amplitude enhancement of the ssVEP reflects heightened visuocortical activation in response to a stimulus, which has been demonstrated to be modulated both by bottom-up sources of signal salience [24] and top-down, task-related processes [25], [26]. The neural sources of the ssVEP have been localized to the primary and extended visual cortex [27], [28], with strong contributions from retinotopic areas, but also from cortices higher in the visual hierarchy [29]. In experiments on differential aversive conditioning, ssVEP and ssVEF responses (its magnetic relative measured by magnetencephalography) were found to be reliably enhanced for CS+ compared to CS- cues [9], [10], [30], [31], [32].

Based on the literature as reviewed above, we examined the hypothesis that affective CS cues elicit larger sensory responses compared to neutral CS cues, following differential social conditioning with pictures of affective and neutral gestures as US. Based on differential amygdala activity findings in social conditioning and larger motivational relevance of negative gestures of insult, we further explored whether visual cortex activity was also higher for CS cues paired with negative compared to CS cues paired with positive gestures.

## Methods

### Participants

Twenty undergraduate students (11 females, mean age *M* = 20.8, *SD* = 2.6 years) from the University of Würzburg with normal or corrected-to-normal vision participated in this study for course credits. All participants were screened for personal and family history of photic epilepsy. Nineteen participants were right-handed, one participant left handed. The institutional review board at the University of Würzburg approved all experimental procedures; all participants provided written informed consent.

### Stimuli

**Figure 1:**
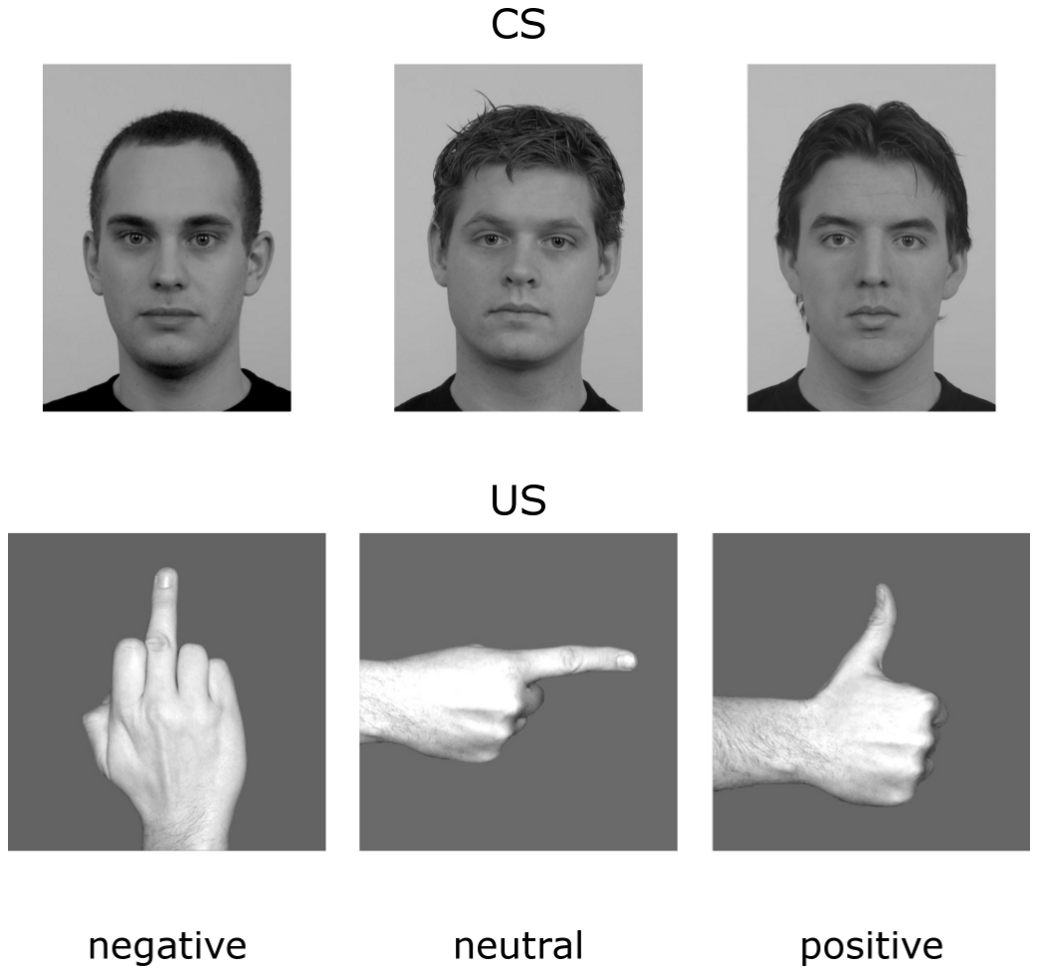
Three male neutral faces served as CS stimuli (upper panel). The affective hand gestures (middle finger, thumbs-up, point gesture) served as US for the differential conditioning procedure (lower panel).

The conditioned stimuli (CS) consisted of pictures of 3 male faces taken from the Radboud Faces database [33], which were converted to grey-scale, adjusted for brightness, luminance and contrast, and presented using Presentation (Neurobehavioral Systems, Inc., Albany, CA, USA). Only male faces were used as it has been shown that male faces seem to be more efficient in fear conditioning and to elicit stronger responses in both men and women, for a review see [34]. The CS cues were delivered for 5000 ms in a flickering mode in front of a uniform gray background at a frequency of 12 Hz in order to elicit the ssVEP. The unconditioned stimuli (US) were pictures of unpleasant, neutral, and pleasant hand gestures [17], [19], which were presented in the conditioning phase only, immediately at offset of the CS faces for 500 ms. Pictures used as CS and US are given in Figure 1.

### Design and Procedure

The experiment contained three blocks (habituation, acquisition, extinction), each consisting of 60 trials (three faces, each presented 20 times) resulting in 180 total trials. In the habituation and extinction phase, faces were presented without any pairings with the US. In the acquisition phase, each face was paired with one of the three hand gestures such that the picture of the respective hand gesture immediately followed the 5000 ms presentation of the face stimulus. The combination of faces and hand gestures was counter-balanced across participants. The order of the stimuli within each block was pseudo-randomized such that no more than two of the same faces ever occurred consecutively during the different phases. After providing written informed consent and initial screening to rule out photic epilepsy/seizures, participants were seated in a sound-attenuated, dimly lit testing room where the electroencephalogram (EEG) sensor net was applied. Participants were instructed that they would view flickering faces of three different individuals, which would at some point during the experiment be combined with pictures of hand gestures. Participants were not informed of a specific relation between CSs and the US. Each picture was displayed centrally on a 19-inch computer monitor (resolution = 1280×1024 pixel) with a vertical refresh rate of 60 Hz, located approximately 80 cm in front of the participant, resulting in a picture presentation with a visual angle of 4.2° horizontally and 5.9° vertically. Each CS was presented on the screen for 5000 ms, with inter-trial intervals varying between 2000 and 3000 ms. Participants were asked to rate each CS stimulus for hedonic valence and arousal after each phase (Habituation, Acquisition, Extinction) using a computer-based version of the Self-Assessment Manikin Scale SAM [35]. The SAM is a language-free instrument for rating hedonic valence and consists of a graphic figure representing nine levels of pleasure/displeasure. Contingency awareness was also assessed using an online analogue scale, in which participants were to indicate the probability of the face to be paired with one of the three US. The purpose of the contingency rating was to determine whether participants successfully learned the CS-US pairing rule. The contingency ratings were obtained immediately after the conditioning phase. After the three experimental phases, participants were asked to rate the US stimuli for affective valence and arousal using the SAM scales.

### EEG Data Recording

EEG was recorded continuously from 129 electrodes using an Electrical Geodesics (EGI) high-density EEG system and digitized at a rate of 250 Hz, using Cz as a recording reference. Impedances were kept below 50 kΩ, as recommended for the Electrical Geodesics high input-impedance amplifiers. All channels were filtered on-line with 0.1 and 100-Hz and 50 Hz notch filter.

### EEG Data Reduction and Data Analysis

Offline EEG analyses were implemented using the ElectroMagnetoEncephalography toolbox for MATLAB [36]. Epochs of 600 ms pre-stimulus and 5600 ms post-stimulus onset were extracted offline. Data were filtered using a 40-Hz low-pass (45 dB/octave, 12^th^ order Butterworth) filter. Artifact rejection was performed following the procedure proposed by Junghöfer, Elbert, Tucker, and Rockstroh [37]. This procedure creates distributions of statistical indices of data quality and allows to identify bad channels and trials, with the latter being discarded and the former being interpolated from the full channel set. In a subsequent step, data was re-referenced to average reference, and artifact-free trials were averaged for each subject according to experimental conditions. Trials were rejected when more than 20 channels out of 129 were outlying as per the statistical parameters used for artifact identification: the mean absolute (rectified) amplitude; the variability over time points; and the maximum first order derivate (gradient). Using this method, 74% of the trials were retained. A minimum number of 3 trials per condition were retained. The number of artifact-free trials did not differ between conditions per phase.

**Figure 2:**
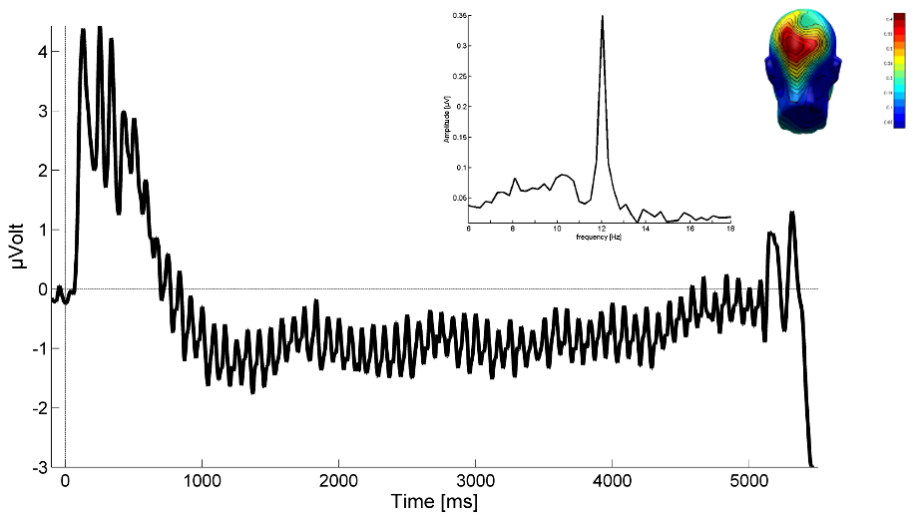
The grand mean steady-state visually evoked potential averaged across all participants and conditions, recorded from a medial occipital electrode (Oz) is presented. The ssVEP in the present study contains the driving frequency (12 Hz) of the face stimulus, as shown by the frequency domain representation (middle inlay) of the same signal (Fast Fourier Transformation of the ssVEP in a time segment between 200 and 5,000 ms. The right inlay shows the mean scalp topography of the very frequency over visual cortical areas.

The artifact-free ssVEP epochs were averaged, and the time-varying amplitude of the ssVEP signal was then extracted by means of Hilbert transform on the time-domain averaged ssVEP data [9]. To this end, data were first bandpass-filtered with a 12^th^ order Butterworth filter having a width of .5-Hz (48 dB/octave), around the target frequency of 12 Hz. To achieve high time resolution, instantaneous amplitudes of the band-pass filtered signal were computed using the Hilbert function implemented in MATLAB. The Hilbert transformation possesses high temporal resolution for indexing rapid changes in ssVEP amplitude. The absolute value of Hilbert transform corresponds to the envelope of the averaged waveform [38]. Figure 2 depicts the steady-state visually evoked potential (averaged across conditions and participants) in the time domain, demonstrating the onset of the oscillatory visuocortical response at the driving frequency (12 Hz) and its frequency spectrum as derived from FFT.

### Statistical Analysis

**Figure 3:**
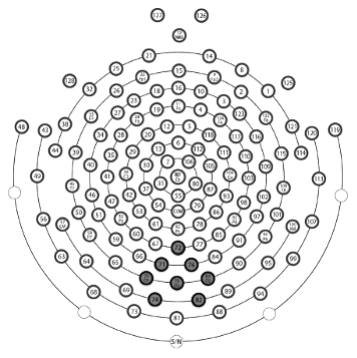
Layout of the dense electrode array. Locations of the electrodes grouped for regional means (used for statistical analysis) are in gray. Sensor #75 corresponds to Oz of the International 10–20 System.

As was seen in previous work with centrally presented stimuli [9], [28], [39], [40], [41], [42], amplitudes of the ssVEPs were most pronounced at electrode locations near the medial occipital electrode Oz, over the occipital pole. Thus, to test conditioning-induced changes in visuo-cortical responses to the different CS, the ssVEP activity was averaged across 8 medial occipital sensors including Oz in the International 10/20 System (EGI sensors 70, 71, 72, 74, 75, 76, 82, 83; see Figure 3).

Mean ssVEP amplitudes (100–4900 ms) were analyzed by means of repeated-measures analysis of variance (ANOVAs). The ANOVA contained the following within-subjects factors: Phase (Habituation, Acquisition, Extinction), and CS-Type (CS^neg^ vs. CS^neu^ vs. CS^pos^). To investigate whether cortical activation differed across picture presentation time [9], an additional ANOVA analysis was carried out using two time windows of the ssVEP amplitudes (100–2500 ms and 2501–4900 ms), consequently including the factor time (early vs. late) as an additional within-subject factor. SAM ratings for valence and arousal were averaged for each stimulus and phase, and submitted to separate mixed-model ANOVAs, containing the within-subjects factors Phase and CS type. The Mauchly's test of sphericity was used to test for violations of this assumption and wherever relevant, the Greenhouse-Geisser corrected results are provided with uncorrected degrees of freedom, corrected F and p values [43].

## Results

### Electrocortical activity (ssVEPs)

**Figure 4:**
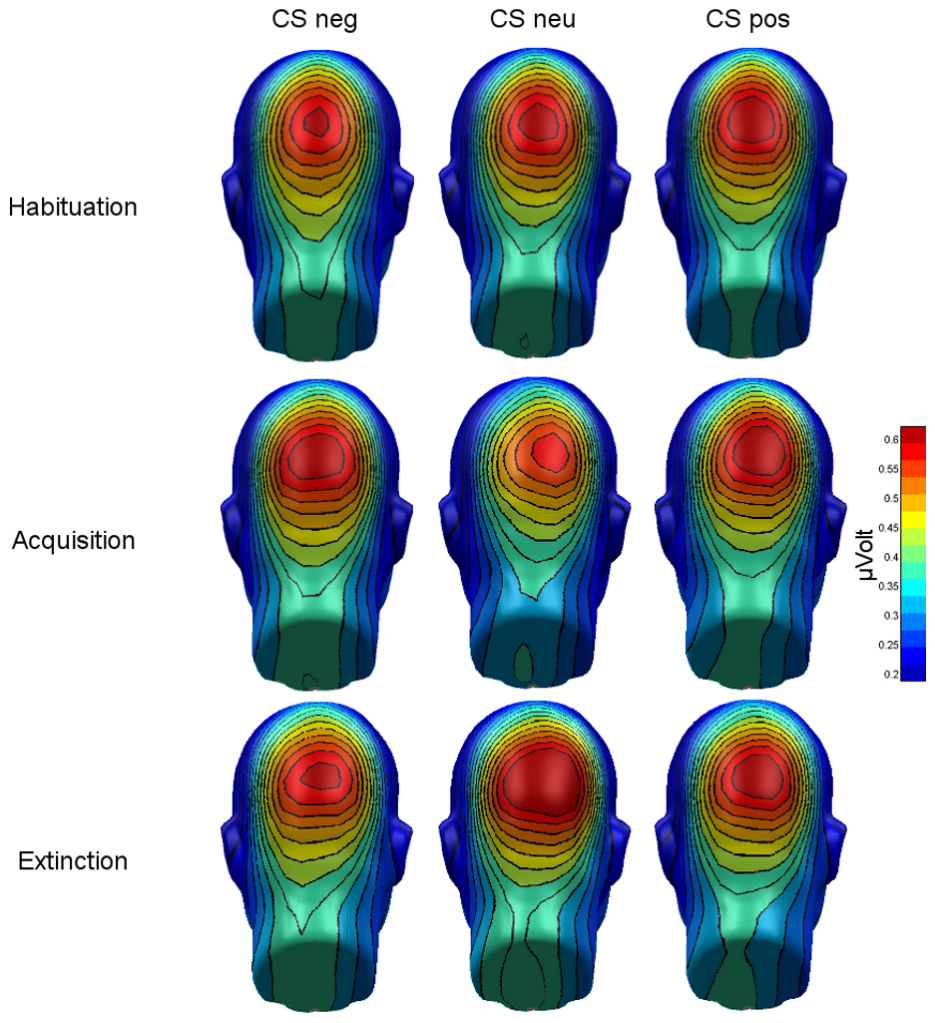
Mean scalp topographies of ssVEP amplitudes (100–4,900 ms) elicited by CS^neg^, CS^neu^, and CS^pos^ faces in during the three phases of the experiment (habituation, acquisition, extinction).

**Figure 5:**
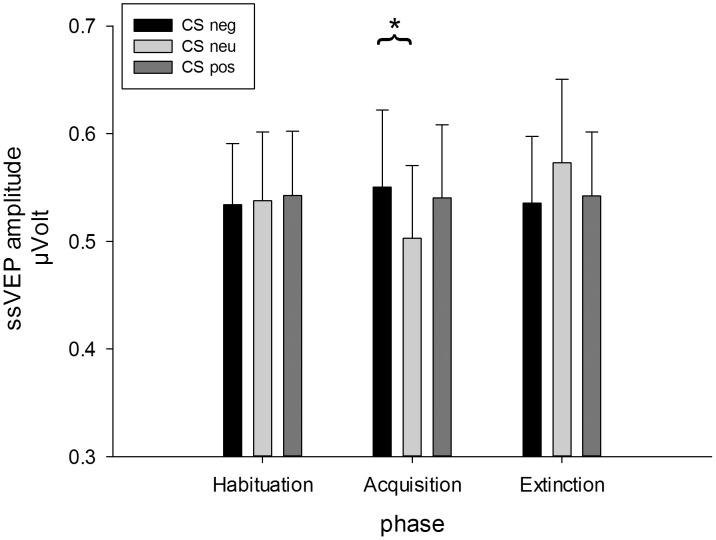
Mean ssVEP amplitudes (100–4,900 ms) +SEM evoked by CS^neg^, CS^neu^, and CS^pos^ faces in during the three phases of the experiment (habituation, acquisition, extinction). Amplitudes are averaged across a medial-occipital cluster comprising Oz and its 7 nearest neighbors.

The ANOVA on the mean amplitudes across the whole viewing time revealed a significant interaction of phase and CS type, *F*(4,76) = 2.88, GG-ε = .63, *p* = .045, η_p_^2^ = .13. (see Figure 4). Separate ANOVAs per phase revealed significant modulations of the ssVEP amplitude for the conditioning phase, only, *F*(2,38) = 3.82, *p* = .031, η_p_^2^ = .17. Planned contrasts showed that CS^neg^ faces evoked larger ssVEP amplitudes compared to CS^neu^ faces, *t*(19) = 2.73, *p* = .013 (Bonferroni-corrected *p* = .017), but CS^pos^ faces compared to CS^neu^ faces elicited only marginally larger amplitudes, *t*(19) = 1.93, *p* = .069 (Bonferroni-corrected *p* = .017). No differences emerged between CS^neg^ and CS^pos^ faces (Figure 5).

The analysis of the time course of the ssVEP amplitude including earlier and later time windows (100–2500 ms and 2501–4900 ms) did not find any interaction including the factor time, but a significant main effect of time, *F*(1,19) = 8.91, *p* = .008, η_p_^2^ = .32, with higher amplitudes in the first compared to the second time window. Additionally, the Phase x CS type interaction was significant, *F*(4,76) = 2.88, GG-ε = .61, *p* = .045, η_p_^2^ = .13.

### Affective Ratings

**Figure 6:**
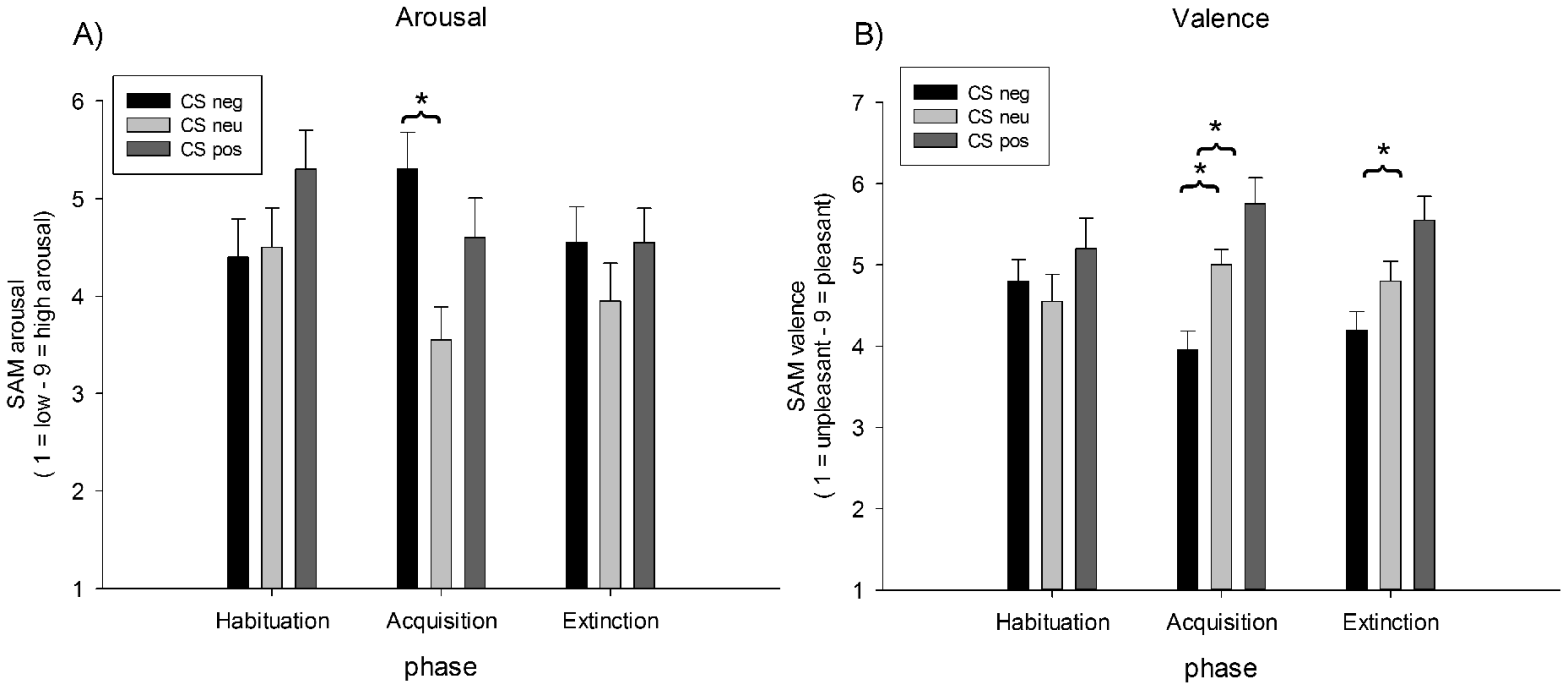
Mean SAM affective ratings collected after each phase. A) Mean arousal ratings (+SEM) of CS^neg^, CS^neu^, and CS^pos^ faces, B) mean valence ratings (+SEM) of CS^neg^, CS^neu^, and CS^pos^ faces.

As expected, arousal and valence ratings changed across the three phases of the experiment depending on the CS type, as the interaction of Phase X CS Type indicated, *F*(4,76) = 3.35, *p* = .014, η_p_^2^ = .15, and *F*(4,76) = 2.81, *p* = .031, η_p_^2^ = .13, respectively (see Figure 6).

For both ratings, also a significant main effect of CS type was observed: arousal ratings, *F*(2,38) = 4.60, GG-€ = .77, *p* = .026, η_p_^2^ = .20; valence ratings: *F*(2,38) = 7.96, *p* = .001, η_p_^2^ = .30. To follow up on the interaction, separate ANOVAS per phase were run. For arousal ratings it turned out that differences were only significant in the conditioning phase, *F*(2,38) = 7.60, *p* = .002, η_p_^2^ = .29. This was due to CS^neg^ face cues were rated as to be more arousing compared to CS^neu^ faces, *t*(19) = 4.09, *p* = .001, whereas the comparison of CS^pos^ and CS^neu^ just missed significance, *t*(19) = 2.24, *p* = .037 (Bonferroni-corrected *p* = .017). For valence ratings, separate ANOVAS per phase revealed significant differences between CS types after the conditioning phase, *F*(2,38) = 12.78, *p*<.001, η_p_^2^ = .40, and the extinction phase, *F*(2,38) = 6.32, GG-€ = .78, *p* = .009, η_p_^2^ = .25. Post-hoc *t*-tests showed that after conditioning, CS^neg^ cues were rated as to be more unpleasant compared to CS^neu^ and CS^pos^, *t*(19) = 3.67, *p* = .002, and *t*(19) = 4.56, *p*<.001 (Bonferroni-corrected *p* = .017). After extinction, only the difference between CS^neg^ and CS^pos^ cues was still significant, *t*(19) = 4.76, *p*<.001 (Bonferroni-corrected *p* = .017).

### US ratings

The analysis of the US ratings revealed that the different gestures which served as US during conditioning were rated as differentially arousing as expected, *F*(2,38) = 6.01, *p* = .005, η_p_^2^ = .24. The middle finger gesture (*M* = 5.80, *SD* = 1.74) was rated as more arousing than the point gesture (*M* = 4.15, *SD* = 1.76), *t*(19) = 3.38, *p* = .003, whereas the arousal rating of the thumbs-up gesture (M = 5.40, SD = 1.54) was only marginally higher than the neutral point gesture, *t*(19) = 5.93, *p* = .024 (Bonferroni-corrected *p* = .017). No difference emerged between middle finger and thumbs-up gesture, *t*(19) = 1.05, *p* = .31. With regard to valence, ratings were also modulated by type of pictures, *F*(2,38) = 85.21, *p*<.001, η_p_^2^ = .82. As expected, the insult gesture (*M* = 2.90, *SD* = 1.12) was rated more unpleasant, whereas thumbs-up gesture was rated as more pleasant (*M* = 7.30, *SD* = 0.80) than the neutral point gesture (*M* = 4.55, *SD* = 0.95), *t*(19) = 4.44, *p*<.001, and *t*(19) = 9.23, *p*<.001.

### Contingency ratings

The analysis of correctly identified contingencies per category did not reveal any differences between CS types, *F*(2,38) = 1.48, *p* = .241, η_p_^2^ = .07. Faces were correctly identified as CS^neg^ in 97.5%, as CS^neu^ in 98.0%, and as CS^pos^ in 99.5% of cases.

## Discussion

Faces associated with negative social cues (raised middle fingers) elicited stronger mass neuronal responses within the visual cortex compared to faces associated with neutral social signals. No differences were found between face-evoked cortical activity in response to faces that indicated negative compared to positive social consequences, however, the difference between neutrally and positively associated faces was only small. Affective ratings confirm these findings, but also demonstrate longer-lasting effects in explicit ratings, as differences were still observable after the extinction phase. Altogether, the findings suggest that response gain in local cortical population activity is modulated by the acquired social and motivational significance of the faces.

The enhanced electrocortical activation in response to faces predictive of negative social signals indicates that social conditioning alters visuocortical processing in a similar manner as more conventional aversive conditioning in which gratings were associated with aversive sounds or electrical shocks [9], [10], [30], [31], [32], [41]. These adaptive changes in function of early visual cortices leads to augmented sensory gain and consequently enhanced processing of CS+ related features [44]. This change in sensory processing during social fear acquisition may be due to transient plasticity of sensory cortical networks [45]. Most likely, this short-term plasticity related to the individual learning history is due to re-entrant modulations of visual areas both by sub-cortical areas such as the amygdala as well as top-down influences of the fronto-parietal attention network. This corroborates findings which demonstrated that the amygdala shows elevated responses to socially conditioned stimuli [13], [14], [16]. Findings of conditioned responses in the lateral amygdala [46] and thalamus [47] preceding those that are observed in the primary sensory cortices support the notion that subcortical centers are necessary for the induction of sustained fear-related plasticity in the cortex. The amygdala can serve to enhance visual cortex activity given extensive bidirectional connectivity between amygdalar nuclei and multiple stages of visual hierarchy known to exist in the brains of human and non-human animals [48]. However, endogenous processes within sensory cortices may also underlie some transient forms of plasticity [49] in sensory cortical areas.

Notably, the amplification of sensory processing in response to socially conditioned faces bears striking similarities to the enhanced processing of visual cues which are inherently threatening such as aversive pictures or threatening faces [50], [51]. Thus, sensory cortices seem to preferentially react to threatening information regardless of their threat values to be acquired by associated learning processes or inherent due to preparedness mechanisms of phylogenetic origin [52]. This observation is also in line with assumptions that sensory cortical networks are rather characterized as being highly adaptive and continuously shaped by the organism's learning history than just holding invariant representations of the external world [53], [54]. Thus, features that are especially predictive of negative outcomes due to a learning history lead to enhanced sensory gain [41]. Future research may compare differences in the processing of inherent and acquired threat cues directly to further shed light on the nature of the development of anxiety and anxiety disorders [55].

The nature of the US in the present study (affective symbolic gestures) points at the notion that nonverbal socio-communicative signals may serve as cues in social learning experience. Given the high emotional significance of particularly the aversive raised middle finger gesture [17], [19], it seems plausible to regard the current paradigm as an excellent model for real-life situations in which subjects are exposed to social stress and form their impressions based on the social consequences they experienced with the very person. The result of the strongest learning effect with the negative gesture (raised middle finger) is in line with enhanced early cortical activity observed in the processing of this gesture of insult [19], which is most likely due to its immediate association with social threat and need of urgent action [17]. In line with other studies [13], [14], [16] using more ecologically valid US such as human voices, faces, verbal feedback, our results confirm that an emotional nonverbal gesture is sufficient to cause conditioning and modulate responses in the visual cortex. It has to be noted that this effect is observed although the US (picture) clearly is much less intense than conventional US such as electrical stimuli. Altogether, the present results make the paradigm of social conditioning with nonverbal gestures an interesting avenue for research on social learning and altered social conditioning in social anxiety, where enhanced sensitivity to social conditioning is assumed [15]. It has to be noted that the current paradigm is also a particular form of evaluative conditioning, in which pairings of positive or negative stimuli (US) with neutral stimuli (CS) induce the learning of evaluative reactions to the target stimuli [56]. Thus, originally neutral faces adopt the evaluative color of the US gestures with which they have been paired previously. Further research needs to clarify whether social conditioning is a different phenomenon such that social CS and US lead to stronger associative learning compared to evaluative conditioning by the easier association of two stimuli social in nature, i.e. a face and nonverbal gesture versus a face and aversive picture of a snake, for example. One may assume that social conditioning as presented here leads to stronger effects for implicit measures (such as visuocortical responses), which have been found to be rather weak in more conventional evaluative conditioning paradigms [57].

The present findings also add to the notion that the perception and evaluation of faces is critically dependent on the context in which faces appear [58], [59]. For example, it has been shown in several studies where faces were combined with emotional bodies that a congruent affective value of the body helps the identification of the facial expression [60], [61]. An important difference between these studies and the present study is that in our study the face gains affective value in a learning procedure through repeated association with affective gestures, whereas in the studies mentioned above the faces themselves were inherently affective (e.g., angry or happy facial expressions), but this recognition of this affective value was facilitated by congruent affective body postures. Nevertheless, both lines of research point to the notion that the affective value of a face is influenced by contextual factors such as concurrent affective body postures or learned associations between faces and affective gestures.

It also has to be noted that the faces paired with positive affective gestures also showed slightly higher electrocortical signal amplitudes compared to neutral faces, albeit non-significant. This effect which may have missed statistical significance due to statistical power, possibly indicates that the observed contextual modulation of face perception may not be entirely exclusive to negative USs, but that faces associated with positive outcomes may also attract more attention compared to neutrally associated faces. In accordance with this notion, the positive hand gesture has consistently been shown to also receive priority processing which, however, is considerably reduced as compared to the higher arousing negative middle finger gesture [17], [19].

### Conclusion

The current study introduces a social conditioning paradigm incorporating socially relevant US of nonverbal affective gestures. The ssVEP in response to the CS faces as well as subjective ratings indicate that faces combined with aversive hand gestures (raised middle finger) are perceived as more negative and arousing, which is also accompanied by elevated visuocortical processing. Such results highlight the importance of using ecologically valid US in conditioning when social learning processes in impression formation are the main area of interest. Moreover, the current paradigm offers a potential means for the study of social learning and its modulation in psychiatric disorders with deficits in social information processing such as in social anxiety disorder and autism.
